# Fighting-related injuries do not affect mate choice in the giant mesquite bug, *Pachylis neocalifornicus*

**DOI:** 10.1093/beheco/araf024

**Published:** 2025-03-23

**Authors:** Lauren A Cirino, Isaac McEvoy, Juliana L Swanson, Zachary Emberts

**Affiliations:** Department of Integrative Biology, Oklahoma State University, 501 Life Sciences West, Stillwater, OK 74078, United States; Department of Biological Sciences, University of Mary Washington, Jepson Science Center 1301 College Avenue, Fredericksburg, VA, 22401, United States; Department of Integrative Biology, Oklahoma State University, 501 Life Sciences West, Stillwater, OK 74078, United States; Department of Integrative Biology, Oklahoma State University, 501 Life Sciences West, Stillwater, OK 74078, United States; Department of Integrative Biology, Oklahoma State University, 501 Life Sciences West, Stillwater, OK 74078, United States

**Keywords:** female mate choice, injury, insects, male-male combat, sexually-selected weapons

## Abstract

Females often choose high-quality mates as they may confer benefits to the female. One way male quality may decline is due to the injuries that they can acquire during male-male combat. Females might assess males based on injury since injuries place energic demands on the body that could reduce their reproductive output. Thus, females might make mating decisions based on whether males have acquired these fighting-related injuries. Here, we tested this injury-mediated female mate choice hypothesis using the giant mesquite bug, *Pachylis neocalifornicus* (Hemiptera: Coreidae). This hypothesis predicts that females will choose uninjured males over injured males. We simulated non-lethal injuries that males could acquire during male-male contests and assayed mate choice. We compared mate choice of the injury group to a control group and found that fighting-related injuries did not affect mate choice. However, females were more likely to mate with males that had large sexually selected weapons while males were more likely to make mating attempts with large-bodied females. Additionally, the smaller a male’s weaponry the more quickly they initiated their mating attempts. Our results do not support the injury-mediated mate choice hypothesis. Instead, they reveal that other factors besides fighting-related injuries appear to have a larger role in determining mating behavior patterns in this species.

## Teaser

Fighting-related injuries may reduce male quality and, thus, decrease mating success. Here, we examined whether female mate choice is influenced by male injuries in the giant mesquite bug. We predicted that females would choose uninjured over injured males since uninjured males may be of higher quality. We found that fighting-related injuries did not affect mate choice. Instead, females chose males with larger sexually selected weapons, a trait that may more accurately signal male quality.

## Introduction

Across many animal taxa males from the same species will compete with one another over access to females (Andersson, 1994; Wiens and Tuschhoff, 2020). Losers of these fights are often excluded from mating opportunities while winners will frequently copulate (Wong and Candolin, 2005; Hunt et al., 2009). However, just because a winner gets the chance to mate does not necessarily mean they will. Females have a choice. Indeed, females often choose to mate with dominant males as dominance generally correlates with higher quality (Qvarnström and Forsgren, 1998; Wong and Candolin, 2005). Mating with high-quality males may confer direct or indirect benefits to the female (Andersson, 1994; Qvarnström and Forsgren, 1998). In some cases, however, dominance may be an unreliable indicator of male quality or breeding site. Thus, females may use traits outside of dominance status to determine quality (Qvarnström and Forsgren, 1998). Examples of this include female cockroaches that choose beta males because they have more of the attractive male pheromone compared to alphas (Moore and Moore, 1999). Additionally, large male dragonflies that usually win intrasexual competitions over smaller males do not necessarily copulate since females choose males with an intermediate body size (Moore, 1990). Thus, males that are considered high quality, and in turn chosen by females, are not always dominant males. Because of this disparity between these sexual selection mechanisms (ie male-male combat and female mate choice), it is important to also consider female mate choice in species that engage in male-male combat.

Male quality, or the condition upon which females select mates, can change due to a variety of factors including diet, age, and infection status (eg Jones and Elgar, 2004; Rantala and Kortet, 2004; Candolin, 2019). Male condition can also be influenced by injuries acquired during male-male combat. Typical fighting-related injuries can include bite wounds (mice: Brown, 1953; lizards: Lailvaux et al., 2004; fish: Santos and Richard, 1996; Neat et al., 1998; Hsu et al., 2011) and punctures (crabs: Dennenmoser and Christy, 2013; shrimp: Rojas et al., 2012), along with cuts, scrapes, and tears (insect wings: Alcock, 1996; Leong et al., 1993; Rüppell and Hilfert-Rüppell, 2013; frogs: Candaten et al., 2020; primates: Kappeler, 1997; turtles: Keevil et al., 2017; dolphins: Scott et al., 2005; Lee et al., 2019). Fitness costs associated with fighting-related injuries are a critical assumption of fighting persistence models (Parker and Rubenstein 1981; Hammerstein and Parker, 1982; Enquist and Leimar, 1983; Payne, 1998; Lane and Briffa, 2017; Palaoro and Briffa, 2017) and relatively few studies have investigated the validity of this assumption (Emberts, Somjee, et al., 2021). Studies that have investigated this assumption have focused on indirect costs of fighting related injuries (eg metabolism and locomotion; Emberts, Somjee, et al., 2021) as opposed to more direct measures of fitness (eg mating).

It seems reasonable that females may evaluate males based on fighting-related injuries. Injuries acquired in a non-fighting context have been shown to influence reproduction (Harwood et al., 2013; Reavey et al., 2014; Shandilya et al., 2018; Greenway et al., 2023). For example, leg injuries in flies cause reduced mating success compared to intact males (Harwood et al., 2013) while autotomy leg injuries in true bugs increase copulation duration (Greenway et al., 2023). Moreover, injuries decrease beetle fecundity, egg viability (Shandilya et al., 2018), and fertility (Reavey et al., 2014) compared to uninjured males. Since injuries acquired outside of male competition can have negative reproductive consequences, it is likely that injury due to male combat may also have detrimental fitness consequences. Because of these potential fitness consequences, males that are in good condition (ie fewer or no injuries) should be favored by females (ie the injury-mediated female mate choice hypothesis). Alternatively, females may associate injury cues (coupled with male presence) with winners of male-male competition. Contest winners may provide benefits to females that outweigh the costs of fighting injuries. Thus, females may choose to mate with injured males instead.

**Fig. 1.**
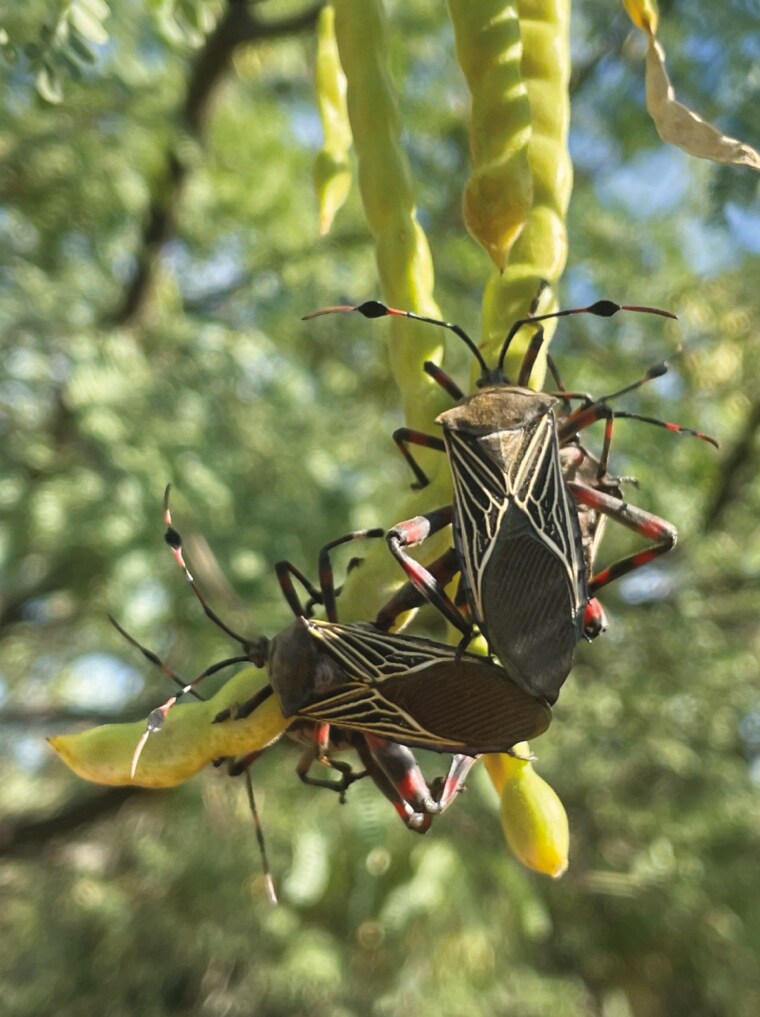
*Pachylis neocalifornicus* mating pair on the fruit of a velvet mesquite tree in Santa Cruz County, Arizona. Photo credit: Lauren A. Cirino.

The giant mesquite bug, *Pachylis neocalifornicus* (Hemiptera: Coreidae) (Fig. 1), provides an excellent opportunity to test the injury-mediated female mate choice hypothesis. Males of this species incur injury through male-male combat that they use to acquire territory and access mates (Emberts, Hwang, et al., 2021; Emberts, Somjee, et al., 2021). Males fight by ventrally aligning with one another and using their hind legs (ie their weapon) to strike and squeeze the dorsal side of their rivals (Emberts and Wiens, 2021; Graham and Emberts, 2023). Their hind legs have tibial spines that can puncture through the wings, that lie flat on the dorsum, and into the abdomen creating metabolically and locomotory costly injuries (Emberts, Somjee, et al., 2021; Graham and Emberts, 2023; McEvoy and Emberts, 2024). Hind leg femur width (a proxy for weapon size) rather than body size best predicts the outcome of fights (Graham and Emberts, 2023). It’s possible that both competing males can acquire injuries in this species, although it is less likely for the winning male (Z. Emberts, personal observation). Thus, even if males win a competition, it is possible that they may still be injured after the fight, making injury status a potentially poor indicator of fighting success.

**Fig. 2.**
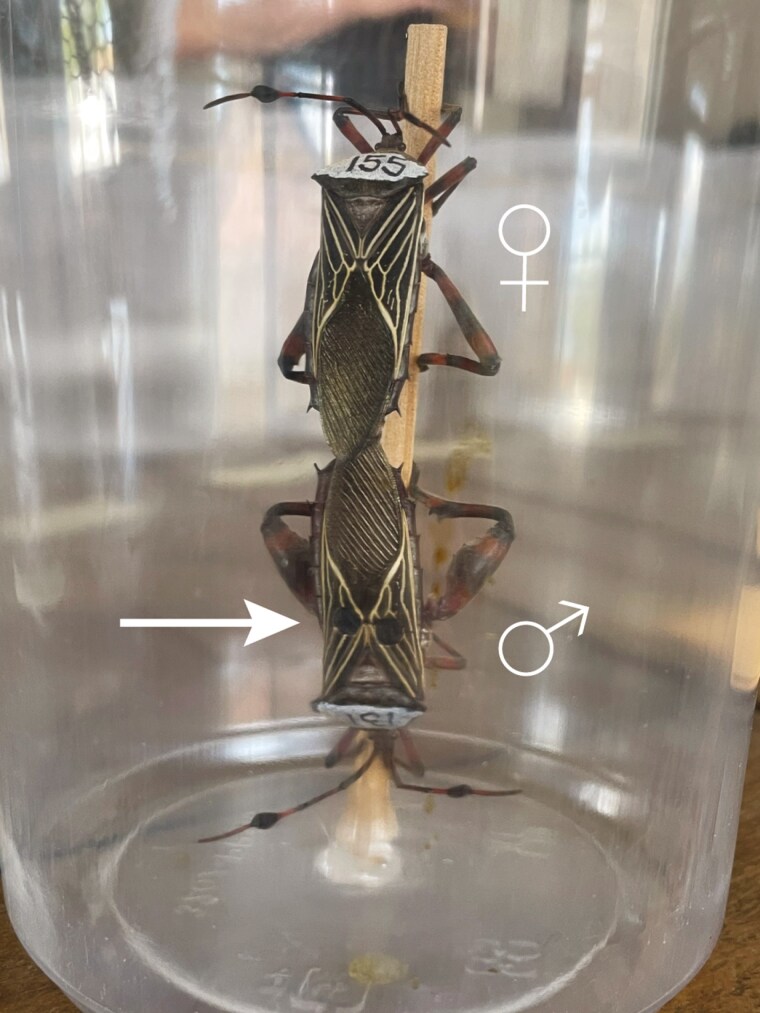
Example of a *Pachylis neocalifornicus* mating pair in copula in the behavioral trial containers. The male in this mating pair was experimentally manipulated to be injured (white arrow) on the forewings. Photo credit: Lauren A. Cirino.

We tested our competing mate choice hypotheses against each other by experimentally injuring *P. neocalifornicus* males to simulate an injury they could acquire during male-male combat (Fig. 2). We then placed one male (injured or uninjured) with a female partner in an isolated environment and noted when males attempted to mate and whether females accepted the attempt (Fig. 2). We expected females to discriminate between males based on their injury status. The injury-mediated female mate choice hypothesis predicts that females will choose uninjured rather than injured males since mating with injured males may have negative reproductive consequences. Alternatively, females may use injury (coupled with male presence) as an indicator of dominance status (ie higher male quality). This hypothesis predicts that females would choose males that are injured over those that are uninjured. Finally, females may not be able to discriminate between males based on their injury status and choose males with or without injuries equally.

## Methods

### Study species

*Pachylis neocalifornicus* feed and breed on velvet mesquite trees (*Prosopis velutina*), specifically on the new foliage and fruits (seed pods). Adults aggregate around these resources in small groups of two to four individuals (Emberts et al., 2023). Groups are commonly composed of one male and one female, and it is rare to see multiple males in a group (Emberts et al., 2023). Indeed, fights occur between males and the winning male holds territory and has access to females while the loser is excluded from mating opportunities (Z. Emberts, personal observation).

### Experimental design

Adult *Pachylis neocalifornicus* were collected by hand in Santa Cruz County, Arizona, in August of 2023 and July of 2024 for behavior trials. Because these bugs were wild caught, their previous fighting and mating experiences were unknown. These insects likely had varied experiences in the wild, so using these animals at this stage allows us to better understand how they will behave in the wild. Sex was determined for all collected individuals and then each were provided with a unique identification number. Identification numbers were written onto the pronotum (region on the dorsal side of the prothorax) of each individual using white paint pens and permanent markers (Fig. 2). Before experimentation began, males and females were kept in sex-specific mesh holding containers (305 mm × 305 mm × 607 mm; L × W × H; with up to 40 individuals per container). The holding containers had fresh cuttings of velvet mesquite (*Prosopis velutina*) for food and a damp paper towel for water. All insects were kept in captivity for less than 36 h before their behavior trials.

Males and females were randomly paired and then pairs were randomly assigned to either the injury or control treatments using a random number generator (n = 80 pairs per treatment). Approximately 30 min before the behavior trials started, the male forewings in the injury treatment (specifically, their corium) were punctured using a 3 mm diameter hole puncher. This method simulated a large, but still biologically relevant, fighting-related injury following Emberts, Hwang et al. (2021). This type of injury is known to have a metabolic cost approximately 30 min after the damage occurs (Emberts, Somjee, et al., 2021). Males in the control treatment were handled in the same way but their forewings were not actually punctured. Once males received their treatment, they were immediately placed into a mating arena consisting of a deli cup (top diameter: 118 mm, bottom diameter: 85 mm, height: 148 mm) that had a wooden dowel fixed in the middle (diameter: 4.7 mm, height: 103 mm) (Fig. 2). This wooden dowel size was selected because it is similar to the diameter of branches that adults are found on (Emberts and Wiens, 2021). After 30 min, females (which were not injured) were added into their respective arenas. “No choice” mating behavior trials were conducted where females can accept the mating attempt made by the provided male or not (ie receptive versus not receptive). These mating situations are very common in the wild as males are most frequently found by themselves or with one female (Emberts et al., 2023). Mating trials began once females were added to the arenas and continued for 3 h. All trials were conducted indoors and in temperatures that ranged between 28 and 29 °C.

Up to 12 mating pairs were monitored at one time. Concurrent behavior trials could be conducted because mating behaviors are stereotypic and have long periods of stasis. Males will approach females and mount them dorsoventrally. Males will then tilt their abdomen to make genital contact. If a female is receptive and accepts the male, the female will allow intromission (Figs 1 and 2). Mating attempts by males (ie male mating effort and choice) and female acceptance or rejection (ie female mate choice) were recorded. A mating attempt was recorded if the male climbed on the female’s dorsum and paused. Females can reject males by running away or closing their genital plates. An acceptance was recorded if the male made sustained genital contact after intromission.

Finer scale behavioral metrics of male mating effort and female mate choice were also quantified. Male mating effort metrics include the latency to the first mating attempt and the total amount of time that males were mounted on females. Latency to the first mating attempt was measured by quantifying the amount of time it took for males to approach and mount a female. The time was calculated by subtracting the time that the male mounted from the time that the behavioral trial started. There were three pairs in which time was not recorded at the first mount, so they were not included in the analysis of this behavioral variable (see Statistical Analyses). The total amount of time that males were mounted on females was quantified by summing the time for each mount that a male made. Time mounted began once a male mounted a female dorsoventrally and ended when either the male was rejected or a copulation began. Another metric of female mate choice that was quantified was latency to copulation. This behavior was calculated by subtracting the time of copulation from the time that males mounted females. Finally, the amount of time that copulations lasted between mating pairs was recorded. Copulation time concluded when the mating pair genitals were no longer touching or the end of the behavior trial. All but six mating pairs were still mating at the end of the 3-h behavior trials. There was also an error in time notation of one of the pairs for time to copulation, so this pair was not included in the analysis (see Statistical Analyses).

Insects were cold euthanized at the end of the behavioral trials. Pronotal width of all insects (ie body size) and hind leg femur width of males (ie weapon size) were then measured to the nearest micrometer at the widest points on those body parts. Body size is an important factor in mate choice (Jennions and Petrie, 1997; Bonduriansky, 2001; Hunt et al. 2009) and weapon size is important to dominance status in this species (Graham and Emberts, 2023). Insects were photographed using a digital camera (Canon EOS 7D, Canon, Tokyo, Japan). The right hind leg was removed from the body by gently gripping the leg close to the trochanter-femur joint and twisting. This hind leg was then placed next to the body, which was dorsal side up, on a light box. This positioning allowed for pronotum width ((a proxy for body size; Procter et al., 2012; Emberts et al., 2020) and hind leg femur width (a proxy for weapon size; Graham and Emberts, 2023) measurements to be taken for males. Pronotal width for both females and males and hind femur width for males were measured using ImageJ software (version 1.53t; Abràmoff et al., 2004).

### Statistical analyses

All statistical analyses were conducted in R (v. 4.4.1; R Core Team 2024). To test whether fighting-related injuries significantly influenced mating behavior and success, we conducted a series of generalized (GLMM) and linear mixed models (LMM; lme4 package v. 1.1-35; Bates et al., 2015) as well as two time-to-event analyses (coxme package v. 2.2-22; Therneau, 2024).

### Male mate choice and mating effort

Our first model investigated whether injury status (injury or control) influenced the likelihood that a male would attempt to mate (ie male mate choice). Mating attempt was a binary variable and simply indicated whether a male mounted the female during the 3-h trial. This model (and all subsequent models) initially included three continuous covariates: female pronotal width (10.082 mm to 14.248 mm), male pronotal width (9.301 mm to 13.770 mm), and male hind femur width (2.507 mm to 5.510 mm). We also included year as a random effect in this model and all models hereafter. After this model was constructed, we removed covariates that were not significant (p > 0.15 following Bursac et al., 2008) in a stepwise fashion starting with the covariates that had the highest p-values. Note that injury status, the main variable of interest, was never removed from any of our models even if it was not significant. This final model ultimately included female pronotal width and injury status (injury: n = 80 pairs, control: n = 80 pairs) as explanatory variables.

In our second analysis, we constructed a Cox proportional hazards model with random effects (time-to-event) to examine males’ latency to mount females. This analysis only included males that made at least one mating attempt (injury: n = 58 pairs, control: n = 53 pairs). We had three less pairs to analyze in this model compared to the next two models because of errors in time notation. Our final model included injury status and male hind femur width.

For our next two analyses, we investigated whether injury status influenced the number of mating attempts (count data using a GLMM with a poisson distribution) and total duration of the mating attempts (GLMM using a negative binomial distribution) (ie male mating effort). Like the time-to-event analysis above, these analyses also only included mating pairs where the male made at least one attempt (injury: n = 59 pairs, control: n = 55 pairs). The final models included injury status and male body size as the explanatory variables for the number of mating attempts model and only injury status as the explanatory variable for the total mount duration model.

### Female mate choice

We examined whether injury status influenced the likelihood that females would accept male mating attempts (ie female receptivity) (GLMM with binary distribution) and the duration of mating (LMM with gaussian distribution). The female receptivity model only included mating pairs where the male made at least one mating attempt (injury: n = 59 pairs; control = 55 pairs). The duration of mating model only included mating pairs in which females accepted a mating attempt (injury: n = 14 pairs; control: n = 13 pairs). All female mate choice models also initially included female pronotal width, male pronotal width, and male hind femur width as covariates and removed the covariates that were not significant from the model in a stepwise fashion. Our final model for female receptivity only included injury status and male hind femur width as explanatory variables, whereas our final model for total mating duration included injury status and female body size as explanatory variables.

Our final analysis examined the time it took for females to accept a male’s mating attempt (ie latency to mate). We constructed a second Cox proportional hazards model with random effects (time-to-event) and the final model included male injury status and female body size as the explanatory variables. We had one less mating pair included in this analysis compared to the previous analysis due to an error in time notation (injury: n = 14 pairs, control: n = 12 pairs).

## Results

**Fig. 3.**
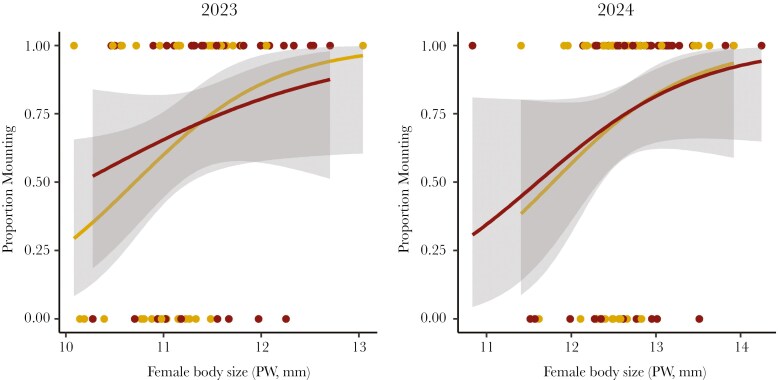
Fighting-related injuries (red) did not influence male mate choice compared to control males (yellow), but males were more likely to attempt to mate with larger females (PW = pronotal width) in both years of the experiment. The shaded gray regions represent 95% confidence intervals.

Males attempted to mate in 114 out of the 160 trials (71%). Among treatments, 59 out of the 80 injured males attempted to mate (74%), while 55 out of the 80 control males attempted to mate (69%). Thus, fighting-related injuries did not influence the likelihood that males would try to mate (GLMM: χ^2^ = 0.181, df = 1, p = 0.670; Fig. 3). Instead, female body size strongly influenced whether a male attempted to mate (χ^2^ = 7.704, df = 1, p = 0.006; Fig. 3), as males were more likely to mount larger females. Male injury also did not influence the latency for males to mount (time-to-event: χ^2^ = 1.422, df = 1, p = 0.233). Instead, males with small hind leg weapons mounted more quickly than males with large hind leg weapons (time-to-event: χ^2^ = 6.073, df = 1, p = 0.014). We also found no evidence to suggest that injury influenced mating effort (total number of mating attempts GLMM: χ^2^ = 0.041, df = 1, p = 0.839; total mating attempt duration GLMM: χ^2^ = 0.127, df = 1, p = 0.721). Injured males that tried to mate made 5 mating attempts totaling 186 s on average, while control males made 4.7 attempts totaling 204 s. Instead, the total number of mating attempts was influenced by male body size as larger males made more mating attempts (GLMM: χ^2^ = 27.192, df = 1, p < 0.001).

**Fig. 4.**
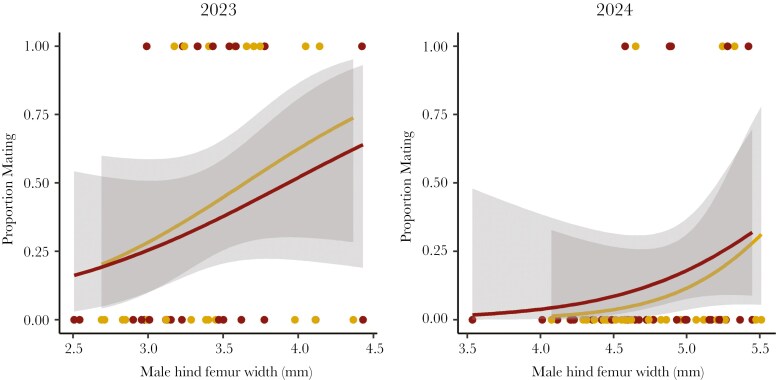
Females were more likely to mate with males that possessed large weapons (ie hind femur width) while male injury status (injured males = red; uninjured males = yellow) did not affect female mate choice. The shaded gray regions represent 95% confidence intervals.

We also found that fighting-related injuries acquired by males do not influence female mating decisions (GLMM: χ^2^ = 0.005, df = 1, p = 0.945; Fig. 4). Females were just as likely to mate with injured males (14 out of 59 females or 24%) as they were with uninjured males (13 out of 55 females or 24%). However, we found that females were more likely to mate with males that had large hind leg weapons compared to their smaller weapon counterparts (GLMM: χ^2^ = 4.669, df = 1, p = 0.031; Fig. 4). Injury status also did not influence the latency to mate (time-to-event: χ^2^ = 0.149, df = 1, p = 0.699) nor did latency to mate differ based on female body size (time-to-event: χ^2^ = 3.356, df = 1, p = 0.067). The total time spent mating was also not affected by injury status (LMM: χ^2^ = 0.007, df = 1, p = 0.935) or female body size (LMM: χ^2^ = 3.805, df = 1, p = 0.051).

## Discussion

Male giant mesquite bugs incur injuries when they engage in male-male combat (Emberts, Hwang, et al., 2021) which are assumed to be costly and influence their fighting decisions (Parker and Rubenstein 1981; Hammerstein and Parker, 1982; Enquist and Leimar, 1983; Payne, 1998; Lane and Briffa, 2017; Palaoro and Briffa, 2017). Indeed, the indirect fitness costs that result from these injuries include increased metabolic rate and reduced flight performance (Emberts, Somjee, et al., 2021). Here, we tested a direct fitness consequence of fighting-related injuries, the injury-mediated female mate choice hypothesis, where females are more likely to choose mates that are uninjured. We found that there was no effect of these injuries on female mate choice. Instead, we found that females are more likely to mate with males with large hind legs (ie the weapon used in male-male combat). We also found that males are more likely to attempt to mate with large females, a pattern found in many other insect species (Bonduriansky, 2001). Finally, we found that larger males put in more mating effort (ie number of mating attempts) than smaller males and males with smaller hind leg weapons are quicker to mount females than males with larger hind leg weapons.

At first glance, our null female mate choice result in relation to fighting injuries may be surprising. Other non-fighting related injuries do affect reproduction (Harwood et al., 2013; Reavey et al., 2014; Shandilya et al., 2018). Foreleg amputation in male flies, for example, decreased mating success compared to intact males (Harwood et al., 2013). Female flies could likely sense a leg amputation as males mount females by placing all their legs on the dorsal side of the female (Harwood et al., 2013). Therefore, females could use this as a signal of male fly health and reject them (Harwood et al., 2013). However, the females in our study could not tactilely or visually detect wing injury since this injury is on the dorsal side of the body. Further, it is possible that these injuries also do not give off olfactory cues that females can detect, at least within the 3-h trials we conducted in this study. Thus, these females likely use other means outside of injury to discriminate between males.

In a distantly related coreid, *Narnia femorata,* non-fighting related injuries (ie hind leg autotomy) increased copulation duration (Greenway et al. 2023). We did not expect similar mating behavior results to those found in Greenway et al. (2023) since injuries incurred in these two studies differ dramatically. The autotomy injury experienced by the coreid species used in Greenway et al. (2023) were self-induced at a pre-determined breakage point while our study simulated injuries that an opponent would inflict. Autotomy related injuries generally heal quickly and thus the immune response is often muted compared to injuries incurred by wounding arbitrary places on the body (Slos et al., 2009; Yang et al., 2018). Thus, we expected that there would be more reproductive costs associated with injuries that place males in a more immunocompromised state.

Yet, we found no reproductive costs based on male injury status in our study. Although the lack of female mate choice in relationship to injury was similar to results found in the Greenway et al. (2023) study, the total copulation duration results differed. We found no effect of injury on copulation duration, while Greenway et al. (2023) found that autotomized males prolonged copulation compared to intact males. This might be because *P. neocalifornicus* engage in copulations which last for hours (Z. Emberts, personal observation) and our study ended with most mating pairs still in copula. Thus, we were unable to get accurate mating duration estimates in this study. Alternatively, it is possible that we obtained different results because the injuries are located on different parts of the body. In our study, males experienced a wing injury that would likely not have future fighting related consequences. *P. neocalifornicus* males would still be able to engage in fights using their hind leg weapon if they incurred wing injuries. Whereas males in Greenway et al. (2023) experienced hind leg loss (ie a damaged weapon) and are more likely to lose future fights (Emberts et al., 2018). Thus, the mating behavior strategy may need to change for the coreids in the Greenway et al. (2023) study compared to *P. neocalifornicus* as it might have negative consequences on their direct fitness if they do not.

Although female mate choice remains unaffected by fighting-related injuries acquired by males, negative reproductive consequences beyond pre-copulatory mate choice may still occur. In studies examining non-fighting related injuries, beetles have reduced female fecundity and viability of offspring (Shandilya et al., 2018). Female fertility can also suffer when females mate with injured males (Reavey et al., 2014). Since fighting-related injuries in male giant mesquite bugs incur metabolic costs (Emberts, Somjee, et al., 2021), it is possible that injured males could have reduced reproductive output due to a reproduction-immune tradeoff (Sheldon and Verhulst, 1996). But this direct fitness consequence of fighting-related injuries on reproductive output has yet to be tested in the giant mesquite bug. Moreover, given that wing injuries reduce flight performance (Emberts, Somjee, et al., 2021), it is also possible that fighting-related injuries in this species diminishes a male’s capacity to locate a mate, potentially reducing his reproductive success. This potential reproductive cost, and others, should be explored in future studies.

Injury status did not influence mating behavior in this species, but weapon size did. Since fighting injuries can occur in both winners and losers, male weapons may be a more reliable indicator of male quality due to their high condition dependence (Emlen et al., 2012). Indeed, the size of the hind leg weapon of *P. neocalifornicus*, and not body size, is a good predictor of dominance status (Graham and Emberts, 2023) which may indicate a high-quality mate (Berglund et al., 1996). Other studies have found similar results where females co-opt the weapons that males use to engage in male-male combat to determine their choice of mate (Latruffe et al., 1999; Morina et al., 2018). For example, male fiddler crabs (*Uca tangeri*) with larger claws are more likely to win contests than males with smaller claws and these large-clawed males are also preferred by females (Latruffe et al., 1999). Female deer also prefer males with larger horns than males with smaller horns, a weapon that also increases their likelihood of dominance in fights (Morina et al., 2018). Weapon size might be an easy visual cue for females to detect since hind legs are large, conspicuous, and correlated with body size (Emberts et al. 2023). Thus, females will still reap the benefits of mating with large males if they use hind leg weapons as a cue to mate (eg Côte and Hunte 1989; Savalli and Fox 1998). Hind leg weapons, however, may not be a visual trait that giant mesquite bug females use in mate choice. Larger male hind legs may enable males to better grasp and orient themselves and/or the female during mating attempts which may result in increased female receptivity. Regardless of the reason, the selective pressures applied by female mate choice and male-male combat appear to be acting in concert with one another in this species.

We also found that male giant mesquite bugs are more likely to attempt to mate with large females regardless of injury status. This mating pattern has been observed in several insect species (Bonduriansky, 2001) including another coreid species (Gillespie et al., 2014; Cirino, 2023). Females with large bodies are generally more fecund than small females (Insects: Bonduriansky, 2001; other members of Coreidae: Nishida, 1994; Wilner et al., 2020; Cirino et al., 2022) and mating with large females will likely result in more reproductive success for the mating male.

Males with small hind leg weapons initiated their mating attempts more quickly. Males with small weapons may alter their mating behavior (compared to males with large weapons) to capitalize on the opportunity to potentially mate with an unguarded female since they are unlikely to win fights and gain mating opportunities when males with larger weapons are present (Parker, 1974; Emlen 2008; Emberts et al., 2023; Graham and Emberts, 2023). Indeed, males from other species that have small or no weapons modify their behaviors to gain mating opportunities. An example of this includes hornless *Onthophagus* male beetles that sneak copulations with females compared to their large weapon fighter male counterparts (Emlen, 1997). Ground-nesting male bees with small mandible weapons also sneak copulations compared to males with large weapons (Danforth, 1991). Although implementing a sneaking strategy when weapons are small is a much more drastic behavioral change than a reduction in latency to mate, these behavioral changes may be due to similar fitness constraints. If small weapon males do not change their mating tactic, they may not achieve mating success which dramatically influences their fitness.

Male mating behavior was also influenced by male body size. Larger males invested more effort into the number of mating attempts (ie courtship) than smaller males. This investment in courtship may have occurred because reproductive effort is energetically costly and large males have more available resources to do so (eg Shine and Mason, 2005; Rahman et al., 2013). Smaller males may need to be more discriminatory in how much they invest in courtship because their chances of mating are lower than larger males (eg Côte and Hunte, 1989). Thus, small males may invest less than large males in courtship so that they can optimize their limited resources for survival and future matings (Cordts and Partridge, 1996).

While the number of mating attempts was influenced by male body size, mating attempt duration was not. Mating attempt duration, another metric of male mating effort, was similar among all males in our study. The reason that there are differences between the two male mating effort metrics (ie mating attempt duration and number of mating attempts) we examined in this study might be because of differences in energetic investment. After males made mating attempts, they would frequently stay mounted atop females and not move. Thus, mating attempt duration likely does not provide us with an accurate picture of the energy invested into courting a female. Mating attempt number, however, requires sudden and swift movement of males. Males quickly grab females with their legs and then scramble to mount them as the female usually moves during this process. Thus, the number of mating attempts may be more indicative of the energy investment males put into female courtship.

Female mate choice can be a powerful form of selection, especially when working in concert with male-male competition (Andersson, 1994). Females often choose to mate with dominant males as dominance correlates with high-quality males (Qvarnström and Forsgren, 1998; Wong and Candolin, 2005). High-quality males may confer direct or indirect benefits to the female, which is likely why females choose them (Andersson, 1994; Qvarnström and Forsgren, 1998). Here, we found that fighting-related injuries do not influence female mating decisions of the giant mesquite bug, but hind leg weapon size does. Weapon size might be a more reliable indicator of male quality since males with large weapons are more likely to achieve dominance status (Graham and Emberts, 2023). Further, both competitors can acquire injuries during male-male combat (Z. Emberts, personal observation) making this a less reliable trait for determining quality. Thus, selection to increase hind leg weapon size is likely strong in this species given the fighting and mating benefits that large hind leg weapons provide.

## Data Availability

Analyses reported in this article can be reproduced using the data provided by Cirino et al. (2025).
